# What impact does maths anxiety have on university students?

**DOI:** 10.1186/s40359-021-00537-2

**Published:** 2021-02-25

**Authors:** Eihab Khasawneh, Cameron Gosling, Brett Williams

**Affiliations:** 1 Department of Community Emergency Health and Paramedic Practice, Monash University, Monash University-Peninsula Campus, McMahons Road, Frankston, VIC Australia; 2 Faculty of Applied Medical Sciences, Jordan University of Science and Technology, Irbid, Jordan

**Keywords:** Anxiety, Mathematics anxiety, Barriers, Facilitators, And gender

## Abstract

**Background:**

Maths anxiety is defined as a feeling of tension and apprehension that interferes with maths performance ability, the manipulation of numbers and the solving of mathematical problems in a wide variety of ordinary life and academic situations. Our aim was to identify the facilitators and barriers of maths anxiety in university students.

**Method:**

A scoping review methodology was used in this study. A search of databases including: Cumulative Index of Nursing and Allied Health Literature, Embase, Scopus, PsycInfo, Medline, Education Resources Information Centre, Google Scholar and grey literature. Articles were included if they addressed the maths anxiety concept, identified barriers and facilitators of maths anxiety, had a study population comprised of university students and were in Arabic or English languages.

**Results and discussion:**

After duplicate removal and applying the inclusion criteria, 10 articles were included in this study. Maths anxiety is an issue that effects many disciplines across multiple countries and sectors. The following themes emerged from the included papers: gender, self-awareness, numerical ability, and learning difficulty. The pattern in which gender impacts maths anxiety differs across countries and disciplines. There was a significant positive relationship between students’ maths self-efficacy and maths performance and between maths self-efficacy, drug calculation self-efficacy and drug calculation performance.

**Conclusion:**

Maths anxiety is an issue that effects many disciplines across multiple countries and sectors. Developing anxiety toward maths might be affected by gender; females are more prone to maths anxiety than males. Maths confidence, maths values and self-efficacy are related to self-awareness. Improving these concepts could end up with overcoming maths anxiety and improving performance.

## Introduction

Maths anxiety can be defined as a feeling of tension, apprehension and anxiety that interferes with maths performance ability the manipulation of numbers and the solving of mathematical problems in a wide variety of ordinary life and academic situations [1]. According to Olango [2] maths anxiety consists of an affective, behavioural and cognitive response to a perceived threat to self-esteem that occurs as a response to situations involving mathematics. Maths anxiety, which is rooted in emotional factors, can be differentiated from dyscalculia, which is characterized by a specific cognitive deficit in mathematics [3], in two ways. Firstly, maths anxiety can exist in people who have maths capability even though they may dislike maths. Secondly, maths anxiety has an emotional component which is not the case in dyscalculia [4].

Maths anxiety may occur in all levels of education from primary school to university education. Harari et al. [5] reported that negative reactions and numerical confidence are the most salient dimensions of maths anxiety in a sample of first-grade students. Similar findings were also observed at tertiary levels across multiple disciplines, including health care professions. For example, Roykenes and Larsen [6] studied 116 baccalaureate nursing students and found that there was a negative relationship between previous mathematic likes/dislikes and self-assessment of mathematic ability.

Many factors may contribute to or facilitate the maths anxiety. These factors or facilitators may include teachers, parents, peers and society. Negative experiences of maths learning in classroom or home can lead to maths anxiety [7]. Firstly, the teacher plays important role in making the class more attractive and reducing anxieties. Good maths teachers can create a learning environment in which students have a positive expectation about their learning [8]. Secondly, parents play an important part in developing or reducing the maths anxiety of their children. Parents' behaviours and relations with children are very important in this aspect [7]. By discussing the anxieties and the fears that their children might face, the parents are able to pinpoint any learning problem at early stage [8]. This might prevent the developing of any learning anxieties that the students might face later in life. Moreover, parents’ maths anxiety causes their children to learn less maths over the school year and to have more maths anxiety by the school year's end [9]. Thirdly, peers play important role in facilitating maths anxiety [7]. Peers at any stage of learning may have a negative impact on their colleagues, for example when students might feel inferior in front of their colleagues when they make mistakes [7]. Finally, society can contribute to the development of maths anxiety due to the misconception about mathematics, or maths myths [7].

Maths anxiety has negative impacts on individuals; many students who suffer from mathematics anxiety have little confidence in their ability to do mathematics and tend to take the minimum number of required mathematics courses, which greatly limits their career [10]. Fortunately, certain strategies can act as barriers, or prevent maths anxiety occurring. Uusimaki and Kidman [11] stated that whenever the persons become self-aware of maths anxiety and its consequences, their abilities to overcome it might increase [11]. On the other hand, activity-based learning and online/distance learning may reduce the fear of looking stupid in front of peers [12]. Another strategy is the use of untimed/unassessed (low stakes) tests to reduce the maths anxiety as well as to increase confidence [13]. Relevancy of studying maths can reduce maths anxiety; applying mathematics and statistics to real-life examples rather than pure maths can reduce maths anxiety [13].

Empirical investigations first began on maths anxiety in the 1950s, and Dreger and Alken [14] introduced the concept of maths anxiety to describe students’ attitudinal difficulty with maths. The aim of this study was to identify the facilitators and barriers of maths anxiety in university students using a scoping review methodology.

## Method

A scoping review methodology was used in conducting this study to allow for a greater breadth of literature to be investigated. Scoping reviews identify and map existing literature on a selected subject. This scoping review utilised the Arksey and O’Malley framework which includes six methodological steps: identifying the research question, identifying relevant studies, selecting studies, charting the data, collating, summarising and reporting the results and consulting experts [15]. The scoping approach systematically maps and reviews existing literature on a selected topic [16] including evidence from both peer-reviewed research and the non-peer reviewed literature.

### Identify the research question

After several review iterations, the research team agreed on the question that guided this review: What are the barriers and facilitators of maths anxiety in university students? This question was broad so it could cover a wide literature in different disciplines that allowed a better summary of the available literature.

### Identify relevant studies

A list of search terms was compiled from the available literature and previous research into maths anxiety and students. Suitable Medical Subject Headings (MeSH) terms and free text keywords were identified (Table 1). A search of databases included: Cumulative Index of Nursing and Allied Health Literature (CINHAL), Embase, Scopus, PsycInfo, Medline, ERIC, Trove, Google Scholar and Grey literature. The search involved any related studies from July-2018 backward. Studies in Arabic and English languages were filtered from the search yield and the abstracts scanned. The databases search were conducted by one of the researchers (EK). The search yield resulted in 656 records which were exported to EndNote17 referencing for screening.

**Table 1:** Search strategy including the Medical Subject Heading (MeSH) and the keywords

MeSH University students	MeSH Barriers	MeSH Facilitators	MeSH Maths anxiety	
Keyword University students	Keyword Obstacle* inhibitor*, limit*, blockage*, barricade*, impedimen*, drawback*	Keyword Develop*,promote*, arrange*, aid*, support*, reinforce*, contribute*	Keyword Mathematics, mathematical concepts	Keyword Apprehend*, concern*, panic*, psychologic stress*, problem*, phobia*, fear*, stress*

Duplicates and irrelevant studies were removed by one of the researchers (EK) and potentially relevant abstracts were complied. The selection process was conducted at two levels: a title and abstract review and full-text review. The title and abstract of the retrieved studies were independently screened (EK and BW) for inclusion based on predetermined criteria. In the second stage, the selected studies full text of potentially eligible studies were assessed and inclusion confirmed by two of the authors (EK and BW). After removing the duplicates, (EK and BW) conducted the title and abstract review of 656 articles. After applying the inclusion criteria 20 articles resulted. These 20 articles were reviewed by (EK and BW) for the second time which ended in 10 articles to be involved in the scoping review.

### Study selection (Fig. 1)

**Fig. 1:**
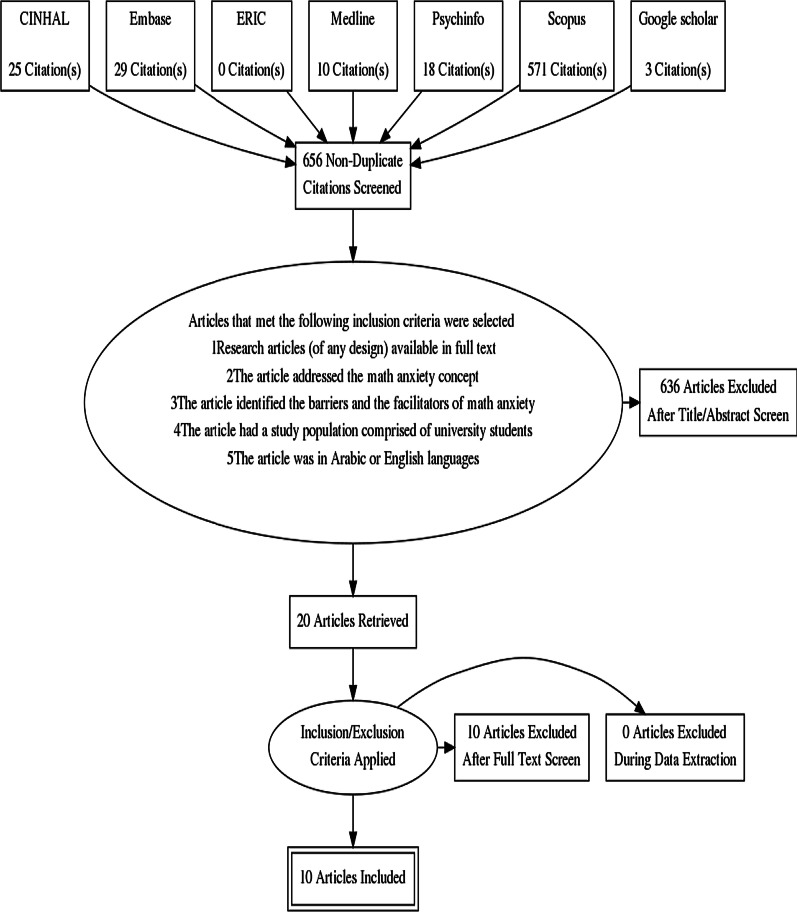
Flow chart of study selection

Articles that met the following inclusion criteria were selected.

1. Research articles (of any design) available in full text.
2. The article addressed the maths anxiety concept.
3. The article identified the barriers and the facilitators of maths anxiety.
4. The article had a study population comprised of university students.
5. The article was in Arabic or English languages.

Articles that are systematic and scoping reviews, abstracts, editorials and letters for editors were excluded.

### Charting the data

This stage allows data extraction from the included studies for more data description. A narrative review method was used to extract the data from each study. Narrative reviews summarise studies from which conclusions can be drawn into more holistic interpretation by the reviewers [17]. The data included: the author and the year of publication, the country the study was conducted in, the study design or type, the sample size, results and the theme emerges from the study (Table 2). Four themes emerged following full-text review of the 10 included papers, these included: gender, self-awareness, numerical ability and learning difficulties.

**Table 2:** Included studies reporting author and year of publication, country of origin, undergraduate discipline, study design and participant numbers, primary outcomes and scoping review theme(s)

References	Country	Discipline	Design and participants	Outcomes	Theme
Zeidner [22]	Israel	Social science and education students who had undertaken a statistics unit	Cross sectional *n* = 431	Lack of maths foundation and low maths self-esteem reinforce maths anxiety Females have higher statistics test anxiety than males (59.33 > 54.28, t (429) = − 3.29, *p* < 0.001) Statistics anxiety is correlated positively with maths anxiety experienced in high school(r (265) = 0.410, *p* < 0.05	Self-awareness
Hopko et al. [19]	USA	Psychology	Randomised controlled trial *n* = 64	High maths anxious individuals generally exhibited higher error rates on mathematical tasks especially those requiring working memory resources MARS-R mean score = 59.0. Females reported more maths anxiety than males (*t* (812) = 5.53, *p* < 0.001)	Gender
Maloney et al. [28]	Canada	Not reported	Cross sectional *n* = 48	High Maths Anxious (HMA) individuals have less precise representations of numerical magnitude than their Low Maths Anxious (LMA) peers t test on the slopes of the distance effects for HMA and LMA by revealing a steeper numerical distance effect for HMA than for LMA group (46) = 2.0, *p* = 0.05	Gender
McMullan et al. (26)	UK	Nursing	Cross sectional *n* = 229	Strong significant relationship between maths anxiety, self-efficacy and ability in nursing students 93% of the Students who failed the numeracy test and 83% of those who failed the drug calculation test demonstrated signs of maths anxiety (score > 25%) Students who failed numeracy test were significantly more anxious (*t* (216) = 7.04, *p* < 0.001 and less confident (*t* (214) = − 5.77, *p* < .001) in performing numerical calculations and less confident in performing drug calculation (*t* (213) = − 3.42, *p* = 0.001)	Numerical ability
Hunt et al. (20)	UK	Psychology	Cross sectional *n* = 78	There is a significant positive correlation between maths anxiety and fixations, dwell time, and saccades. No significant difference between males and females on self-reported maths anxiety *r* (67) = 0.13, *p* = 0.26. Overall sample mean for maths anxiety 48.42, SD = 13.97	Self-awareness
Jordan et al. [30]	UK	Psychology, nursing and other	Prospective cohort Dyslexia group *n* = 28 Control group *n* = 71	Higher maths anxiety was associated with having a dyslexia diagnosis The predictors of maths anxiety: self-esteem(*r* = − 327, *p* = 0.001),worrying (*r* = .394; *p* < .001), denial(*r* = .238; *p* = 0.018), seeking instrumental support(*r* = .206; *p* = 0.040) and positive reinterpretation(*r* = -.216; *p* = 0.032)	Numerical ability
Liew et al. [18]	USA	Not reported	Cross sectional *n* = 184	Interventions targeting emotion regulation and stress management skills may help individuals to reduce their maths and test anxieties Coefficient of gender on avoidance temperament was significant, higher in females (M = 0.17, SD = 0.80) than in males (M = − 0.41, SD = 0.84)	Gender
Hendy et al. (27)	USA	Mathematics	Cross sectional *n* = 368	Students with low Maths confidence or high maths anxiety might benefit from the maths self-evaluation and self-regulation intervention that is guided by Ramdass and Zimmerman 2008 suggestions The scales related to maths belief that are used in this study: Maths Value Scale (MVS), Maths Confidence Scale (MCS) and Maths Barriers Scale (MBS) The most commonly reported maths belief was Maths Confidence (mean rating = 3.79, SD = 0.90	Learning difficulty
Alves [23]	Portugal	Engineering	Cross sectional *n* = 140	Gender shows no differences in the perceived importance of maths anxiety. The Mann–Whitney test showed no significant differences in anxiety toward maths between male and female students (*U* = 2270.5, *p* = 0.541) No significant differences in self-efficacy between male and female students (*U* = 2110.5, *p* = 0.198)	Gender
Paechter et al. [21]	Austria	Psychology	Prospective cohort *n* = 225	High maths anxious individuals have less precise representations of numerical magnitude than their low maths anxious peers Female students report higher levels of maths anxiety *β* = 0.660 Participants with a higher propensity to experience anxiety in general report higher levels of maths anxiety (*β* = 0.385)	Gender

### Collating, summarising and reporting the results

The data extracted from the included studies are reported in Table 2. The table shows a summary of the selected articles in this scoping review study. It presents data on the different scales used to evaluate the maths anxiety across the different disciplines. Key outcome data from each of the included studies is presented and includes some of the causes or predictors of maths anxiety in university students such as gender and self-efficacy.

### Consultation (optional)

Two experts were contacted for consultation to ensure no new or existing literature was missed; however no new articles were added following this consultation.

## Discussion

Maths anxiety is an issue that effects many disciplines across multiple countries and sectors. Literature analysed in this scoping review spanned disciplines as diverse as education, engineering, health and science while covering diverse geographical locations such as United States (US), Austria, United Kingdom (UK), Israel, Portugal and Canada. The included articles utilised an array of varied study designs, including, cross-sectional, randomised control trial, and prospective cohort studies. The main themes that emerged from this review include gender, self-awareness, numerical ability, and learning difficulty each of these will now be synthesised and discussed.

### Gender

Six articles addressed the gender concept; two American studies, three European and one Israeli study with mixed findings for the role gender plays in maths anxiety. Some of these articles found that gender has a role in maths anxiety [18, 18, 20, 21], while others found there was no significant difference between males and females [20, 22]. For example, a study of female psychology students in the US reported more maths anxiety than males [19] whereas there was no significant difference between males and females in maths anxiety in psychology students reported in the UK [20]. Psychology female students in the US [19] and Austria [21], and social science and education female students in Israel showed more maths anxiety than male students [22]. While in another study there was no significant difference in maths anxiety between males and females in the Portuguese engineering students [23].

The reasons why females frequently report higher maths anxiety than males is not well understood [24]. One explanation might be the different gender socialisation during childhood may differentially affect the anxiety experienced by males and females in certain situations which is known as the sex-role socialization hypothesis [24]. The sex-role socialization hypothesis argues that because mathematics has been traditionally viewed as a male domain, females may be socialised to think of themselves as mathematically incompetent and therefore females may avoid mathematics. When females do participate in mathematical activities they may experience more anxiety than males [24].

The pattern of gender effect on maths anxiety is different among disciplines and countries. In a recent study, Paechter et al. [21] administered the Revised Maths Anxiety Ratings Scale (R-MARS) to 225 psychology students at the University of Graz, Austria. This study showed that there were three antecedents of maths anxiety. Firstly, female gender who reported a higher level of maths anxiety *β* = − 0.660. Secondly, a high proneness to experience anxiety in general report higher levels of maths anxiety *β* = 0.385. Finally, poor grades in maths. According to Paechter et al. [21] maths anxiety is inversely related to maths grades *β* = 0.393. Of the above three factors, female gender was the most strongly related to maths anxiety and is supported by the findings of other studies such as Devine et al. [23]. Developing anxiety toward maths might be effected by gender and highlights a specific area for future empirical work.

### Self-awareness

Self-awareness helps people to manage themselves and improve performances while the opposite is true that lacking self-awareness leads to making the same mistakes repeatedly [25]. Being self-aware enables us to determine our strengths and areas that can be improved [25]. Four studies addressed the self-awareness concept in relation to maths anxiety, one American study, one UK study, one Israeli study and one Portuguese study. Under the self-awareness theme, a number of other subthemes emerged including self-efficacy, maths confidence, maths value, maths barriers and performance. McMullan et al. [26] developed a Drug Calculations Self-Efficacy Scale that measured critical skills of medication calculations (dose of liquid oral drugs, solid drugs, injections, percentage solutions and infusion and drip rates). McMullan et al. [26] reported that there was a significant positive correlation between students’ maths self-efficacy and maths performance and between maths self-efficacy, drug calculation self-efficacy and drug calculation performance. Low level of maths anxiety was demonstrated by 10% of the students, medium level by 70% and high level by 20% of the students. McMullan et al. [26] also noted that numerical skills can be improved by remedial approaches as lectures, study groups, workshops and computer assisted instructions [27]. The authors suggested that the lectures should be more student-directed not only didactic in nature. Study groups increase the cooperation and encourage students to exchange and clarify information leading to improve the self-efficacy.

Maths confidence, maths value and maths barriers are related to maths behaviour and performance. Hendy et al. [28] studied maths behaviours in 368 university maths students. They reported maths behaviours (attending class, doing homework, reading textbooks and asking for help) at week 8 of the 15 week-semester using self-reported questionnaires. The aim of their study was identify the subclasses of maths beliefs and their role in maths behaviours. The most commonly reported maths belief was maths confidence (mean rating = 3.79, SD = 0.90). This study reported that students with low maths confidence or high maths anxiety might benefit from the maths self-evaluation and self-regulation interventions. These interventions utilised suggestions which include: maths skills are learnable not innate, assessing current skills and believing in their development abilities, teaching student the specific strategies to solve maths problems and keeping self-regulatory records to track development in overcoming maths anxiety. These interventions may be used in overcoming maths anxiety. This study outlined the approach to develop interventional teaching methods that can be applied to students or course curriculum to help in reducing maths anxiety. Self-awareness might determine the person’s areas of strength that might help future career selection. Self-efficacy, maths confidence and values, maths barriers and performance are factors that related to self-awareness. Assessing these factors can determine the methods of improving self-awareness which may end in overcoming maths anxiety.

### Numerical ability

Two articles addressed the numerical ability concept [25, 2]. In their efforts to understand the origin of maths anxiety, Maloney et al. [29] investigated the processing of symbolic magnitude by high and low maths anxious individuals. They reported that high maths anxious individuals have less precise representations of numerical magnitude than their low maths anxious peers. Two experiments were performed on 48 undergraduate students in the University of Waterloo. A single Arabic digit in 18-font Arial font was presented at fixation. Numbers ranged from 1–4 to from 6–9. The participants were told to identify whether the number above five or below it. This study revealed that high maths anxious individuals have a less precise representation of numerical magnitude than the low maths anxious individuals. The results suggest that maths anxiety is associated with low level numerical deficits that compromise the development of higher level mathematical skills.

On the other hand, McMullan et al. [26] reported that numerical ability and maths anxiety are the main personal factors that might influence drug calculation ability in nursing students. The numerical ability test (NAT), used by McMullan et al. [26], is comprised of 15 questions that covered calculation operations like multiplication, addition, fraction, subtraction, percentage, decimals and conversion. McMullan et al. [26] reported that both numerical ability and drug calculation abilities of the participants (229 UK nursing students) were poor which might have been to an over-reliance on using calculators or not having adequate maths education in the past. Improving numerical ability and reducing maths anxiety can be achieved through teaching in a supportive environment using multiple teaching strategies that address the needs of all students and not being didactic [26]. Examples of these strategies include: accept and encourage students creative thinking, tolerate dissent, encourage students to trust their judgments, emphasise that everyone is capable of creativity, and serve as a stimulus for creative thinking through brainstorming and modelling [30].

### Learning difficulty

Australian surveys have indicated that 10 to 16 per cent of students are perceived by their teachers to have learning difficulties according to Learning Difficulty Australia (LDA) (2012). Within the population of students with learning difficulties, there is a smaller subset of students who show persistent and long-lasting learning impairments and these are identified as students with a learning disability. It is estimated that approximately 4 per cent of Australian students have a learning disability (LDA 2012).

In this scoping review, one UK study addressed this concept, comparing undergraduate psychology students who represent 71% of the sample and nursing students who represent 14% of the sample who either had dyslexia (*n* = 28) or were assigned to the control group (*n* = 71). In 2014 Jordan et al. [31] reported that students with dyslexia had higher levels of maths anxiety relative to those without [31]. This study showed that significant correlations with maths anxiety were found for self-esteem (*r* = − 0.327; *n* = 99, *p *.001), worrying (*r* = 393; *n* = 99; *p* < 0.001 the denial (*r* = 0.238; *n* = 99; *p* = 0.018, seeking instrumental support (*r* = 0.206; *n* = 99; *p* = 0.040 and positive reinterpretation (*r* = − 0.216; *n* = 99; *p* = 0.032). In addition, this study found that seeking instrumental support served as an indicator of students at high risk of maths anxiety. In explaining variation in maths anxiety. Jordan et al. [31] claimed that 36% of this variation is due to dyslexia, worrying, denial, seeking instrumental support and positive reinterpretation. The limitation of this study is that not all dyslexia cases were disclosed by the students. As long as some of the students with dyslexia are not reported, the generalisation of this study would be limited. This study recommends positive reframing and thought challenging as techniques to overcome difficult emotions and anxiety.

## Limitations and future research

While multiple databases were used in this scoping review, some articles may be missed due to using specific terms in the search strategy. The disciplines covered in this scoping review were psychology, engineering, mathematics and some of the health disciplines such as nursing. Future research might focus on numerical ability and maths anxiety in university students who need maths and calculation in their future careers as engineers and health care professionals.

For example, the relationship between medication and drug calculation errors and maths anxiety in the health care field can be researched. Moreover, the relationship between self-awareness and numerical ability and maths anxiety and their impact on the performance and ability of the university students can be a future research topic. Finally, developing a new teaching package or strategy that reduces maths anxiety can be tested on university students.

## Conclusion

Maths anxiety,which is an issue that affects many disciplines across multiple countries and sectors, is affected by gender, self-awareness, learning difficulties and numerical ability. Maths anxiety and its contributing factors at tertiary education should be researched more in the future addressing interventions and strategies to overcome maths anxiety. Maths anxiety level measuring tools should be used in determining its level among university students.

## Data Availability

It is a scoping review and all the articles that are analysed in this review are listed in the references section.

## Abbreviations

CINHAL: Cumulative Index of Nursing and Allied Health Literature
ERIC: Education Resources Information Centre
HMA: High Maths Anxious
LDA: Learning Difficulty Australia
LMA: Low Maths Anxious
MBS: Maths Barrier Scale
MCS: Maths Confidence Scale
MeSH: Medical Subject Headings
MVS: Maths Value Scale
UK: United Kingdom
US: United States
R-MARS: Revised Maths Anxiety Rating Scale
